# Supplementary figures and images for: COVID-19 infection and transmission includes complex sequence diversity
Source: PLoS Genet. 2022 Sep 8;18(9):e1010200. doi: 10.1371/journal.pgen.1010200 (PMC9455841; doi:10.1371/journal.pgen.1010200)

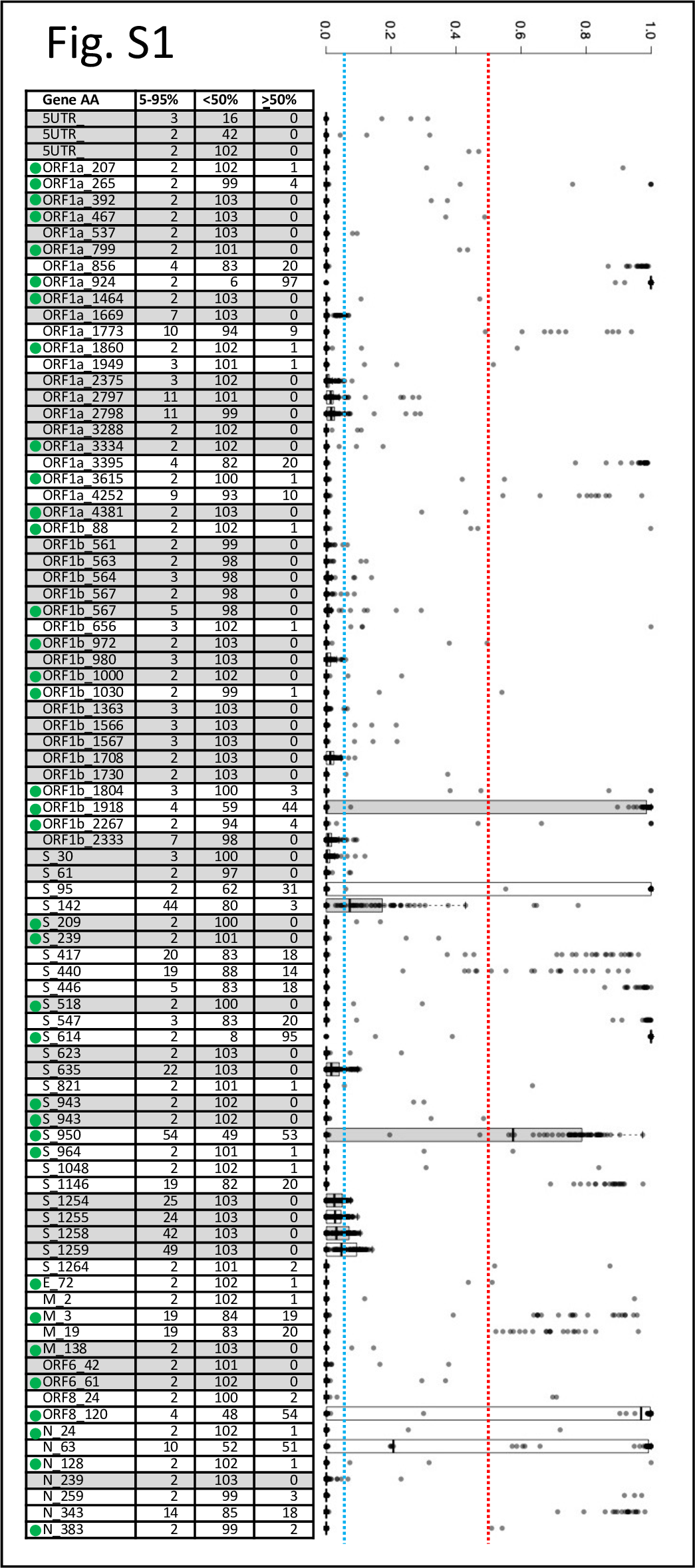

Supplement: S1 Fig — SRA data were accessed from 110 infections reported from 18 different laboratories or consortia as Illumina raw fastq files. These sources included AFRIMS, ATCC, Centre for DNA Fingerprinting and Diagnostics, COG-UK, CZB Covidtracker, Department of Medical Research, Disease Prevention and Control, Federal State Budgetary Scientific Institution Institute Of Experimental Medicine, Gonoshasthaya-RNA Molecular Diagnostics and Research Center, Igenbio Inc, Loma Linda University, National Public Health Laboratory of Malaysia, Network for Genomic Surveillance in South Africa (NGS_SA), The Hashemite University, University of California San Diego Microbiome Init, University of California Irvine, University of Montana, University of Wisconsin–Madison. When available in the SRA metadata, information has been provided in S4 Table regarding specific details on the laboratory or clinic that submitted the patient sample. Box and whisker plots illustrate the distribution of alternate allele frequencies for each mutation (median (bar); 25th and 75th percentiles (bottom and top of box, respectively); 10th and 90th percentiles (bottom and top whiskers, respectively)). The red dashed line at 50% identifies the demarcation at which the alternate allele is recognized and reported as the major consensus nucleotide that characterizes the infecting virus. These 87 mutations were chosen for further analysis because they all showed evidence of biallelic states at greater than the 5% background threshold and were observed in more than 2 infections (blue dashed line). Among these varying positions, 46 mutations did not reach an alternate allele frequency >50% for any of the 110 infections; 41 positions exceeded the 50% threshold for a portion of the 110 infections and would therefore have contributed to characterization of the infecting viral strain. The accompanying table enumerates the number of samples with AAF between 5 and 95%, <50% and >50%. Overall, among the 1,080 alternate alleles dete [file pgen.1010200.s007.tif]

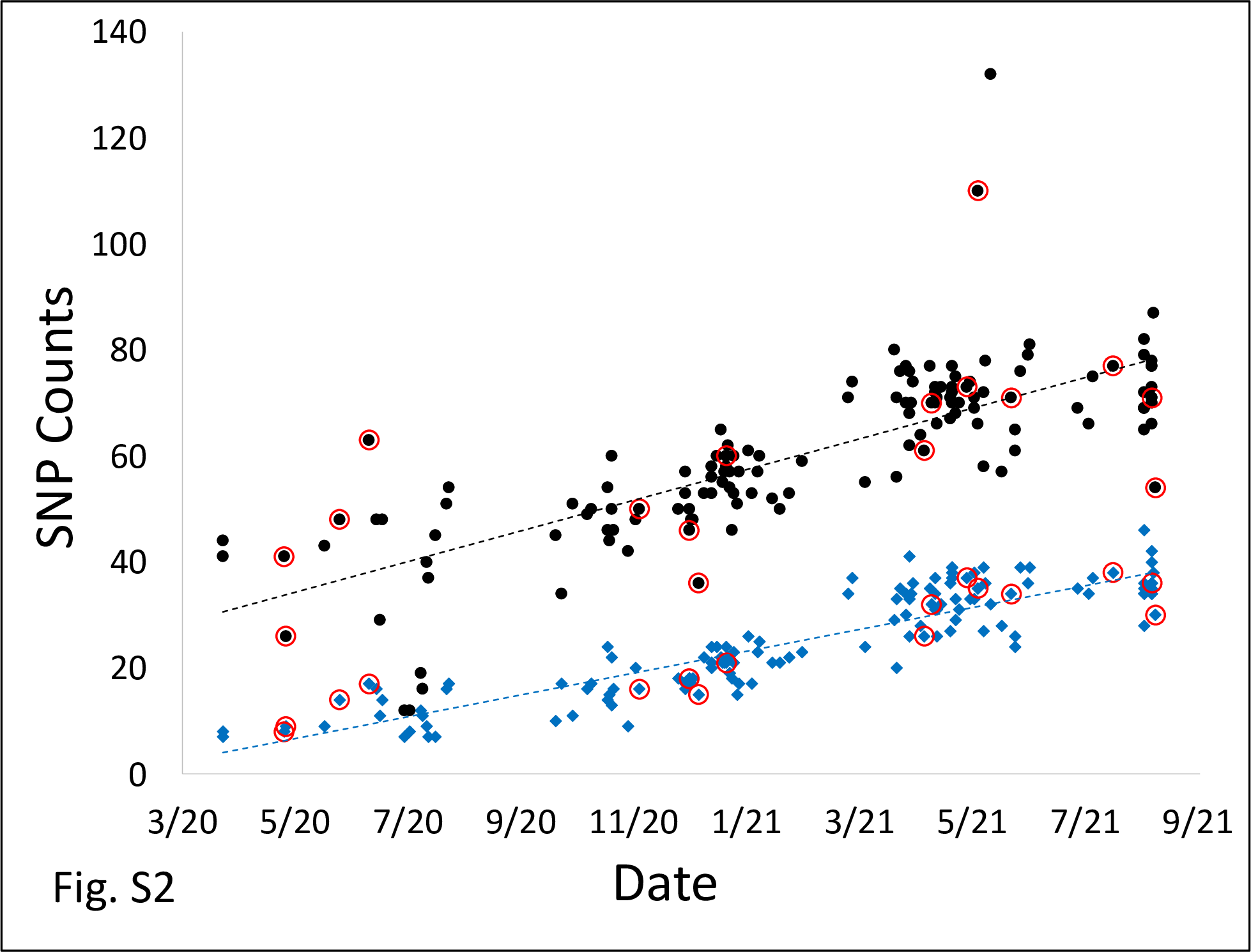

Supplement: S2 Fig — IM- individuals are identified by red circles. Comparisons suggest that there is no difference in the SNVs observed at either threshold. Correlation between the number of alternate alleles identified in each sample and the sample collection date—AAF >5% (black circles), or AAF >50% (blue diamonds); samples were the 140 that were >80% 100x. Significant correlation was observed between an increase in AAFs during the sample collection time period (AAF >5%, Pearson R = 0.76112; AAF >50%, Pearson R = 0.89253; both P-values <0.0001). (TIF) [file pgen.1010200.s008.tif]
